# New Insights into the Genetic Association of Lipids and Lipid‐Modifying Target Genes with Pancreatitis

**DOI:** 10.1002/mco2.70554

**Published:** 2025-12-14

**Authors:** Linbo Yao, Xinmin Yang, Robert Sutton, Qing Xia, Wei Huang

**Affiliations:** ^1^ West China Centre of Excellence for Pancreatitis Institute of Integrated Traditional Chinese and Western Medicine West China‐Liverpool Biomedical Research Centre West China Hospital Sichuan University Chengdu China; ^2^ Liverpool Pancreatitis Research Group Liverpool University Hospitals NHS Foundation Trust and Institute of Systems, Molecular and Integrative Biology University of Liverpool Liverpool UK

1

Pancreatitis is a complex and progressive inflammatory disease of the pancreas, associated with a high risk of morbidity and mortality. It is well known that pancreatitis is a disease progression from the initial episode of acute pancreatitis (AP) to chronic pancreatitis (CP) via recurrent AP (RAP). However, little is known about identifying at risk of recurrences and progression or about pharmacological interventions to prevent these outcomes. While hypertriglyceridemia is a common cause of AP, with pancreatitis secondary to it typically presenting as an episode of AP or RAP, and rarely as CP. While it has been clearly established that the risk of AP correlates positively with triglyceride (TG) levels [1], and that familial chylomicronemia syndrome is extensively documented as a cause of pancreatitis spanning AP, RAP, and CP [2], a comprehensive genetic investigation of these causal links is still warranted. Furthermore, there is no clear casual effect evidence for lipid‐lowering drugs (i.e., statins or fibrates) in reducing the likelihood of pancreatitis.

Therefore, we performed Mendelian randomization (MR) analysis to assess the causal associations between lipid levels and AP, CP, alcohol‐induced AP and CP (AAP and ACP) using data from the FinnGen Consortium. Drug‐target MR and colocalization analyses were further conducted to investigate the potential impact of 15 lipid‐modifying targets on these pancreatitis subtypes. To ensure the validity of instrumental variables, a positive control analysis was conducted in parallel to test causal effect of selected druggable targets on coronary artery disease, which has a well‐established causal association (see Supplementary Material for detailed methodology).

In the primary univariable MR analyses, we found that genetically proxied increases in TG levels were associated with a higher risk of all pancreatitis subcategories. In contrast, genetically proxied increases in high‐density lipoprotein cholesterol (HDL‐C) levels were causally associated with a decreased risk of AP, AAP, and CP. To account for potential confounding factors, we performed multivariable MR analyses. Specifically, we adjusted for genetically predicted cholelithiasis in AP and for genetically predicted alcohol consumption and smoking in CP. We also conducted multivariable MR analyses for AAP and ACP after adjusting for genetic liability to alcohol consumption. Our results were highly consistent across both univariable and multivariable analyses, showing that genetically proxied levels of TG and HDL‐C are causally associated with pancreatitis, whereas no significant association was found between either total cholesterol or low‐density lipoprotein cholesterol and any pancreatitis subcategory.

In the drug‐target MR analyses, we attempted to evaluate effects of 15 lipid‐modifying targets on pancreatitis, namely, *HMGCR*, *NPC1L1*, *PCSK9*, *APOB*, *LDLR*, *ABCG5*, *ABCG8*, *ACLY*, *ANGPTL3*, *MTTP*, *PPARA*, *PPARD*, *PPARG*, *APOC3*, and *LPL*. For TG (Table 1), higher genetically proxied lipid levels mediated by *APOB* increased the risk of CP (odds ratio [OR] = 2.057, 95% confidence interval [CI] 1.290–3.280, *p* = 0.002) and ACP (OR = 2.236, 95% CI 1.163–4.297, *p* = 0.016). Likewise, a potential association of *APOC3* with CP (OR = 1.366, 95% CI 1.111–1.679, *p* = 0.003) and ACP (OR = 1.442, 95% CI 1.080–1.926, *p* = 0.013) was identified. For HDL‐C (Table 1), targeting *PPARG* appeared to decrease the risk of AP (OR = 0.325, 95% CI 0.143–0.740, *p* = 0.007), AAP (OR = 0.045, 95% CI 0.006–0.363, *p* = 0.004), CP (OR = 0.295, 95% CI 0.100–0.871, *p* = 0.027), and ACP (OR = 0.187, 95% CI 0.041–0.852, *p* = 0.030), while targeting *APOB* and *APOC3* appeared to be associated with reduced risk of CP (*APOB*: OR = 0.459, 95% CI 0.262–0.805, *p* = 0.007; *APOC3*: OR = 0.580, 95% CI 0.364–0.924, *p* = 0.022), and ACP (*APOB*: OR = 0.417, 95% CI 0.190–0.916, *p* = 0.029; *APOC3*: OR = 0.412, 95% CI 0.214–0.972, *p* = 0.008). The posterior probability of colocalization between TG and CP in the *APOC3* gene region was 79% conditional on the presence of a causal variant for the outcome (Table 1).

**TABLE 1:** Summary statistics of lipid‐modifying targets used in Mendelian randomization and colocalization analyses.

Exposures	Outcomes	Targets	NSNP in MR	OR (95% CI)	*p* Value	P_pleio	P_heter	NSNP in colocalization	H3 (%)	H4 (%)	H4/(H3 + H4) (%)
TG	CP (FinnGen)	*APOB*	17	2.057 (1.290–3.280)	0.002	0.821	0.936	511	3.226	5.589	63.404
		*APOC3*	29	1.366 (1.111–1.679)	0.003	0.727	0.956	652	2.647	9.932	78.957
	ACP (FinnGen)	*APOB*	17	2.236 (1.163–4.297)	0.016	0.734	0.966	511	2.272	3.643	61.591
		*APOC3*	29	1.442 (1.080–1.926)	0.013	0.470	0.908	652	3.461	4.917	58.689
	CAD	*APOC3*	29	1.253 (1.153–1.361)	0.000	0.946	0.372	675	0.572	94.718	99.400
		*LPL*	40	1.619 (1.470–1.783)	0.000	0.031	0.341	1139	90.492	4.920	5.157
	AP (East Asian)	*LPL*	19	2.800 (1.578–4.966)	0.000	0.234	0.279	689	16.891	33.878	66.729
	AP (European)	*LPL*	41	1.233 (1.001–1.519)	0.049	0.399	0.856	688	1.964	10.360	84.065
	CP (East Asian)	*LPL*	19	0.310 (0.129–0.747)	0.009	0.619	0.074	689	6.947	32.822	82.532
	CP (European)	*APOB*	17	2.192 (1.100–4.370)	0.026	0.932	0.652	338	1.186	6.927	85.384
HDL‐C	AP (FinnGen)	*PPARG*	11	0.325 (0.143–0.740)	0.007	0.082	0.533	978	3.566	16.932	82.601
	AAP (FinnGen)	*PPARG*	11	0.045 (0.006–0.363)	0.004	0.507	0.728	978	4.926	8.734	63.939
	CP (FinnGen)	*APOB*	12	0.459 (0.262–0.805)	0.007	0.797	0.948	511	3.226	5.566	63.306
		*APOC3*	13	0.580 (0.364–0.924)	0.022	0.194	0.792	652	2.647	9.932	78.958
		*PPARG*	11	0.295 (0.100–0.871)	0.027	0.693	0.831	978	5.856	6.828	53.830
	ACP (FinnGen)	*APOB*	12	0.417 (0.190–0.916)	0.029	0.810	0.953	511	2.271	3.662	61.716
		*APOC3*	13	0.412 (0.214–0.792)	0.008	0.153	0.776	652	3.461	4.917	58.689
		*PPARG*	11	0.187 (0.041–0.852)	0.030	0.652	0.585	978	5.242	11.644	68.958
	CAD	*APOC3*	14	0.729 (0.571–0.931)	0.011	0.004	0.015	675	0.572	94.718	99.400
		*LPL*	38	0.612 (0.558–0.671)	0.000	0.013	0.455	1139	27.799	70.788	71.803
		*PPARG*	11	1.676 (1.083–2.593)	0.020	0.740	0.360	1018	4.765	1.902	28.526
	AP (East Asian)	*LPL*	20	0.339 (0.180–0.640)	0.001	0.124	0.221	689	11.766	53.855	82.070
	CP (East Asian)	*LPL*	20	3.222 (1.188–8.739)	0.022	0.567	0.032	689	9.727	6.279	39.228
	CP (European)	*APOB*	12	0.362 (0.159–0.825)	0.016	0.905	0.964	338	1.185	6.968	85.462

Abbreviations: AP, acute pancreatitis; AAP, alcohol‐induced AP; CP, chronic pancreatitis; ACP, alcohol‐induced CP; CAD, coronary artery disease; OR, odds ratio; CI, confidence interval; TG, triglycerides; HDL‐C, high‐density lipoprotein cholesterol; IVW, inverse variance weighted; NSNP, number of single‐nucleotide polymorphisms; APOB, apolipoprotein B; APOC3, apolipoprotein C3; LPL, lipoprotein lipase; PPARG, peroxisome proliferator‐activated receptor gamma. P_pleio is the *p* value of MR‐Egger intercept; P_heter is the p value of the heterogeneity test using Cochran's *Q* value; H3 (%) is the posterior probability of hypothesis 3 in colocalization analysis; H4 (%) is the posterior probability of hypothesis 4 in colocalization analysis; H4/(H3 + H4) (%) represents the probability of colocalization conditional on the presence of a causal variant for the outcome.

To enhance the robustness of our findings, we conducted replication analyses using independent datasets from AP (UK Biobank), as well as East Asian and European consortia for both AP and CP. Replication of the *APOB*‐mediated effect on TG levels revealed a significant association with CP (European) (OR = 2.192, 95% CI 1.100–4.370, *p* = 0.026) (Table 1), consolidating our primary finding. Although the association for *APOC3* did not reach statistical significance in these replication datasets, the *LPL*‐related TG levels, which lacked a significant association in our primary analysis, demonstrated a clear association with AP in the East Asian (OR = 2.800, 95% CI 1.578–4.966, *p* = 0.000) and European (OR = 1.233, 95% CI 1.001–1.519, *p* = 0.049) datasets (Table 1). This newly confirmed association with *LPL* provides crucial evidence supporting its established clinical link with AP.

The drug‐target analyses reinforce our core findings by supporting the causal role of specific lipid metabolism pathways in pancreatitis. These analyses collectively establish distinct causal mechanisms through which *APOC3*, *APOB*, and *LPL* contribute to pancreatitis risk. First, APOC3 elevates systemic concentrations of TG‐rich lipoproteins by inhibiting LPL, the principal enzyme for TG clearance. Second, APOB levels serve as a direct proxy for the number of circulating TG‐rich lipoprotein particles; genetically elevated APOB indicates an increased particle count, thereby intensifying the total TG load delivered to the pancreas.

Recent meta‐analysis and randomized clinical trials of *APOC3* mRNA‐targeted therapeutics, including volanesorsen [3], plozasiran [4], and olezarsen [5], have confirmed their efficacy in both decreasing serum TG levels and lowering the risk of AP. These findings provide strong translational support for our genetic results.

In summary, our findings indicate that higher serum TG and lower serum HDL‐C causally increase the risk of pancreatitis. The drug‐target analyses suggest that targeting *APOB*, *APOC3*, and *LPL* are promising strategies to reduce the risk of multiple forms of pancreatitis, a finding that warrants further investigation.

## Author Contributions

WH and QX are joint senior authors. LY, XY, and WH designed the study. XY, WH, and QX obtained funding and supervised students. XY and WH drafted the manuscript. LY and XY performed the analyses. RS had important intellectual input. WH and RS critically revised the manuscript. All authors read and approved the final version of the article.

## Funding

This study was supported by Sichuan Science and Technology Plan Projects—Key Research and Development Program (awarded to WH); National Nature Science Foundation of China (No. 82304985 to YX; No. 82270672 to WH; No. 82274321 to QX).

## Ethics Statement

The authors have nothing to report.

## Conflicts of Interest

The authors declare no conflicts of interest.

## Supporting information




**Supporting File 1**: mco270554‐sup‐0001‐SuppMat.docx

## Data Availability

All summary statistics analyzed during this MR study are publicly available and sourced from established genome‐wide association study consortia. The primary data for pancreatitis subtypes were sourced from the FinnGen consortium (accessible at https://r10.finngen.fi/), while data for serum lipids, replication analyses, and the coronary artery disease control were retrieved from the Global Lipids Genetics Consortium (https://csg.sph.umich.edu/willer/public/glgc‐lipids2021/) and the IEU GWAS database (https://gwas.mrcieu.ac.uk/). Comprehensive datasets, including univariable/multivariable MR results, external validation data, and summary statistics for lipid‐modifying targets (MR and colocalization), are deposited in Zenodo at https://doi.org/10.5281/zenodo.17707905. No novel software was developed for this study; all analyses were performed using publicly available R packages. The R scripts used to generate the results are available from the corresponding author upon reasonable request.
