# Supplementary material for: New Insights into the Genetic Association of Lipids and Lipid‐Modifying Target Genes with Pancreatitis
Source: MedComm (2020). 2025 Dec 14;6(12):e70554. doi: 10.1002/mco2.70554 (PMC12703042; doi:10.1002/mco2.70554)
Supplement: Supplementary file 1 — Supporting File 1: mco270554‐sup‐0001‐SuppMat.docx [file MCO2-6-e70554-s001.docx]

**New insights into the genetic association of lipids and lipid-modifying target genes with pancreatitis**

**Linbo Yao,^1,#^ Xinmin Yang,^1,#^ Robert Sutton,^2^ Qing Xia,^1,^* Wei Huang,^1,2,^***

^1^West China Pancreatitis Centre for Excellence, Institute of Integrated Traditional Chinese and Western Medicine, West China-Liverpool Biomedical Research Centre, West China Hospital, Sichuan University, Chengdu, People’s Republic of China

^2^Liverpool Pancreatitis Research Group, Liverpool University Hospitals NHS Foundation Trust and Institute of Systems, Molecular and Integrative Biology, University of Liverpool, Liverpool, United Kingdom

***Correspondence:** Wei Huang ([dr_wei_huang@scu.edu.cn](mailto:dr_wei_huang@scu.edu.cn)) and Qing Xia ([xiaqing@medmail.com.cn](mailto:xiaqing@medmail.com.cn))

**^#^** These authors contributed equally to this work.

**Supplemental methods**

This research utilised publicly accessible, deidentified summary data from previous genome-wide association studies (GWAS); ethical clearance was secured for all original studies, with detailed citations provided.

**Genetic association of lipids for pancreatitis and coronary artery disease**

The GWAS summary data for pancreatitis were sourced from the FinnGen consortium. The R10 release (Dec 18, 2023) of the FinnGen consortium data was accessed from https://r10.finngen.fi/^1^. This dataset includes 6,787 cases and 361,641 controls for acute pancreatitis (AP), 3,875 cases and 361,641 controls for chronic pancreatitis (CP), 1,021 cases and 411,160 controls for alcohol-induced acute pancreatitis (AAP), and 1,959 cases and 410,222 controls for alcohol-induced chronic pancreatitis (ACP). Genetic associations with serum lipids were extracted from the latest GWAS conducted by the Global Lipids Genetics Consortium, which encompassed over 1 million individuals of European descent^2^. To mitigate potential bias arising from sample overlap, we employed summary statistics that excluded participants from the FinnGen study. To enhance the robustness of our findings, we conducted replication analyses using independent datasets for AP (UK Biobank) (ID: ukb-b-19388), AP (East Asian) (ID: ebi-a-GCST90018569), AP (European) (ID: ebi-a-GCST90018789), CP (East Asian) (ID: ebi-a-GCST90018601), and CP (European) (ID: ebi-a-GCST90018821) sourced from the IEU GWAS database (<https://gwas.mrcieu.ac.uk/>). Additionally, to ensure the reliability of instrumental variables, we carried out a positive control analysis to estimate the causal effects of the selected druggable targets on coronary artery disease, a firmly established causal association. The genetic associations with coronary artery disease in individuals of European descent were sourced from the IEU GWAS database, which encompassed 141,217 participants. The accession number for this dataset is “ebi-a-GCST003116” ^3^.

**Genetic variant selection**

Among genetic instruments for serum lipids, all single nucleotide polymorphism (SNP) represented common variants with minor allele frequencies > 0.01 and F-statistic > 10. We selected independent genetic variants from GWAS that were significantly associated genome-wide (p < 5 × 10^-8^) with triglycerides, total cholesterol, low-density lipoprotein cholesterol, and high-density lipoprotein cholesterol. These variants were then clumped using the PLINK software, with phase 3 of the 1000 Genomes Project as the reference (linkage disequilibrium (LD) clumping threshold of *r*^2^ < 0.001 with physical distance threshold 10,000 kb).

In the drug-target Mendelian randomisation (MR), we identified SNP within the respective gene loci (±100 kb windows around the target gene region) that exhibited robust associations at the genome-wide significance level (p < 5 × 10^−8^). For example, in the ENSEMBL database (http://www.ensembl.org/index.html), the apolioprotein C3 is positioned at chr11:116,700,422-116,703,788. We extracted SNP located within the region chr11:116,600,422-116,803,788. Given the close proximity of the genes encoding ATP binding cassette subfamily G member 5 (*ABCG5*) and *ABCG8* (*ABCG5*: chr2:44,039,611-44,066,004; *ABCG8*: chr2:44,066,103-44,105,605), variants near these genes were consolidated in our analyses. These variants were subsequently subjected to further clumping at an LD threshold of *r*^2^ < 0.10 with a physical distance threshold of 100 kb, serving as proxies for lipid-modifying drug targets.

**Mendelian randomisation**

The study used the random-effect inverse-variance weighted method as the main approach to estimate the association between genetic risk factors and pancreatitis risk. To ensure the results were reliable, several sensitivity analyses were performed. These included the weighted median method, which can provide unbiased estimates even if some of the genetic variants are invalid, and the MR-Egger method, which helps account for pleiotropy (when a genetic variant affects the outcome through pathways other than the exposure). To account for potential confounding effects, multivariable MR analyses were conducted^4^. Given that gallstone disease is a frequent cause of AP, the analysis for this subtype was adjusted for genetically predicted cholelithiasis using data from the FinnGen consortium (ID: finngen_R10_K11_CHOLELITH). For CP, a multivariable MR analysis was conducted with adjustment for genetically predicted alcohol consumption and smoking. The datasets for these confounders were sourced from the IEU GWAS database, with alcohol consumption data (ID: ieu-b-73) and smoking data (ID: ieu-b-4877). Furthermore, associations between the genetically predicted risk factors and alcohol-induced pancreatitis (AAP and ACP) were assessed using multivariable MR analyses after adjusting for genetic liability to alcohol consumption.

**Colocalisation analysis**

Colocalisation analysis is a widely utilised method for assessing whether a genome-wide signal signifies a shared causal variation between two traits. In our study, we performed a colocalisation analysis to investigate whether the associations between locus and pancreatitis were impacted by LD. This approach is designed to test five hypotheses: H0: no association with either trait; H1: association with trait 1 only; H2: association with trait 2 only; H3: both traits are associated but with distinct causal variants for each (two independent SNP); and H4: both traits are associated with the same shared causal variant (one shared SNP)^5^. Each hypothesis was assigned a posterior probability (H0, PPH1, H2, H3, and H4). For this analysis, we set the prior probabilities for the SNP associations with trait 1 only (p1) at 1 × 10^-4^, the SNP associations with trait 2 only (p2) at 1 × 10^-4^, and the SNP associations with both traits (p12) at 1 × 10^-5^. We considered two signals to strongly support colocalisation if the posterior probability for shared causal variants (PPH4) was greater than or equal to 0.8. Medium colocalisation was indicated if the value of PPH4 fell between 0.5 and 0.8^6^. H4/(H3+H4) represents the probability of colocalisation conditional on the presence of a causal variant for the outcome^7^.

**Statistical analysis**

The primary statistical analyses were conducted using R programming software V.4.3.0 (https://www.r-project.org/). The F-statistic was employed to evaluate the existence of weak instrument bias, calculated as referenced in the study^8^. MR analysis utilised the 'TwoSampleMR' package (V.0.5.7) ^9^. The MVMR package (V.0.4.1)^10^ was used to perform multivariable MR analyses. The coloc package (V.5.2.3)^5^ was used to perform colocalisation analysis.

**References:**

1. Kurki MI, Karjalainen J, Palta P, et al. FinnGen provides genetic insights from a well-phenotyped isolated population. Nature 2023;613:508-518.

2. Graham SE, Clarke SL, Wu KH, et al. The power of genetic diversity in genome-wide association studies of lipids. Nature 2021;600:675-679.

3. Nikpay M, Goel A, Won HH, et al. A comprehensive 1,000 Genomes-based genome-wide association meta-analysis of coronary artery disease. Nat Genet 2015;47:1121-1130.

4. Mao X, Mao S, Sun H, et al. Causal associations between modifiable risk factors and pancreatitis: A comprehensive Mendelian randomization study. Front Immunol 2023;14:1091780.

5. Wallace C. A more accurate method for colocalisation analysis allowing for multiple causal variants. PLoS Genet 2021;17:e1009440.

6. Yuan S, Xu F, Li X, et al. Plasma proteins and onset of type 2 diabetes and diabetic complications: Proteome-wide Mendelian randomization and colocalization analyses. Cell Rep Med 2023;4:101174.

7. Zhao SS, Yiu ZZN, Barton A, et al. Association of Lipid-Lowering Drugs With Risk of Psoriasis: A Mendelian Randomization Study. JAMA Dermatol 2023;159:275-280.

8. Bowden J, Del Greco MF, Minelli C, et al. Improving the accuracy of two-sample summary-data Mendelian randomization: moving beyond the NOME assumption. Int J Epidemiol 2019;48:728-742.

9. Hemani G, Zheng J, Elsworth B, et al. The MR-Base platform supports systematic causal inference across the human phenome. Elife 2018;7.

10. Sanderson E, Spiller W, Bowden J. Testing and correcting for weak and pleiotropic instruments in two-sample multivariable Mendelian randomization. Stat Med 2021;40:5434-5452.
